# Streptavidin-biotin-based directional double Nanobody sandwich ELISA for clinical rapid and sensitive detection of influenza H5N1

**DOI:** 10.1186/s12967-014-0352-5

**Published:** 2014-12-20

**Authors:** Min Zhu, Xue Gong, Yonghong Hu, Weijun Ou, Yakun Wan

**Affiliations:** The Key Laboratory of Developmental Genes and Human Disease, Ministry of Education, Institute of Life Sciences, Southeast University, Nanjing, 210096 P.R. China; Jiangsu Nanobody Engineering and Research Center, Nantong, 226010 P.R. China; State Key Laboratory of Materials-Oriented Chemical Engineering, College of Biotechnology and Pharmaceutical Engineering, Nanjing Tech University, Nanjing, 210009 P.R. China

**Keywords:** Nanobody, Influenza H5N1 virus, Directional, Biotinylation, Streptavidin

## Abstract

**Background:**

Influenza H5N1 is one subtype of the influenza A virus which can infect human bodies and lead to death. Timely diagnosis before its breakout is vital to the human health. The current clinical biochemical diagnosis for influenza virus are still flawed, and the diagnostic kits of H5N1 are mainly based on traditional monoclonal antibodies that hardly meet the requirements for clinical applications. Nanobody is a promising tool for diagnostics and treatment due to its smallest size, high specificity and stability. In this study, a novel Nanobody-based bioassay was developed for rapid, low-cost and sensitive detection of the influenza H5N1 virus.

**Methods:**

Nanobodies specific to H5N1 virus were selected from a VHH library by phage display technology. In this system, the biotinylated Nanobody was directionally captured by streptavidin coated on ELISA plate, which can specifically capture the H5N1 virus. Another Nanobody conjugated with HRP was used as a detector. A novel directional enzyme-linked immunosorbent assay for H5N1 using specific Nanobodies was established and compared to the conventional undirected ELISA assay.

**Results:**

We have successfully constructed a high quality phage display Nanobody library and isolated two Nanobodies against H5N1 with high affinity and specificity. These two Nanobodies were further used to prepare the biosensor detection system. This streptavidin-biotin-based directional double Nanobodies sandwich ELISA for H5N1 detection showed superiority over the commonly undirectional ELISA protocol. The linear range of detection for standards in this immunoassay was approximately 50–1000 ng/mL and the detection limit was 14.1 ng/mL. The average recoveries of H5N1 virus from human serum samples were in the range from 94.58% to 114.51%, with a coefficient of variation less than 6.5%.

**Conclusion:**

Collectively, these results demonstrated that the proposed detection system is an alternative diagnostic tool that enables a rapid, inexpensive, sensitive and specific detection of the influenza virus.

## Background

Influenza virus is a member of the group of single-stranded ribonucleic acid (ssRNA) viruses with negative polarity. It belongs to the family *Orthomyxoviridae* and contains three genera: influenza A, influenza B and influenza C [1,2]. Avian influenza A is a severe infectious disease that occurs and spreads very fast in poultry, wild birds, animals and it is transmissible to humans [3]. According to the antigenicity of their hemagglutinin (HA) and neuraminidase (NA) molecules, the influenza A viruses have been classified into 16 HA subtypes (H1-H16) and 9 NA subtypes (N1-N9) [4,5]. Avian influenza H5N1 virus, a subtype of influenza A virus, has been considered as a potential highly pathogenic virus threatening human health [6]. Since the first human infected with influenza H5N1 in Hong Kong, in 1997 [7], more than 300 cases of death in fifteen countries have been reported by the World Health Organization (http://www.who.int/en/). The most cases of human H5N1 infections were characterized by a severe influenza syndrome, associated with symptoms of fever, cough, short breath and radiological evidence of pneumonia [8]. The influenza H5N1 have seriously impacted both global economy and human health, therefore a rapid and sensitive detection of the H5N1 virus is of great significance.

The rapidly and precisely diagnose the subtype of influenza virus when it breaks out, a variety of methods for detection of the influenza virus have been reported in numerous studies. Virus isolation [9], immunofluorescence [10], polymerase chain reaction (PCR) [11,12], enzyme-linked immunosorbent assay (ELISA) [13] and serological methods are becoming more commonly available in diagnosis. However, these conventional methods are laborious, time-consuming, expensive and require appropriate laboratory facilities. For example, virus isolation was regarded as the gold standard for diagnosis and also indispensable for rapid laboratory confirmation of human influenza in routine, however it often require 5–7 days to test with labor-intensive and long procedures [14]. Another novel method for a rapid and dependable testing of influenza is the use of biosensors. Microgravimetric quartz crystal microbalance (QCM) has been considered as a transducer for virus detection such as influenza A and B viruses [15], but the sensitivity and detection limit of QCM immunosensors are unsatisfactory. Thus, the development of an inexpensive and sensitive method for influenza detections a challenge for scientists all over the world.

The detection of virus particles by antibody-mediated immunoassays is specific and accurate. Monoclonal antibodies (mAbs) against viral proteins were established for the immunological detection of H5N1 influenza virus for research and diagnostic purposes [16]. Nevertheless, traditional monoclonal antibodies used in virus detection need more support costs and they are difficult for massive production. A single variable domain, also called Nanobody® (a trademark of Ablynx NV) or the variable domain of heavy-chain only antibody (VHH), was derived from the heavy chain antibody present in camels, llamas, alpacas and sharks [17,18]. The single domain VHH is the smallest available, functional and intact antigen-binding fragment, only with approximately 15 kDa. Because the VHH prefers to associate with concave-shaped epitopes, it can recognize more inaccessible and cryptic sites, when compared to the conventional antibodies [19]. Several VHHs have been used as new bio-medicine for therapy and evaluated in phase I and II clinical trials by Ablynx (http://www.ablynx.com/). Moreover, Nanobodies are easily expressed in large quantities, and have excellent stability and high affinity with the target antigen [20]. Based on these eminent properties, Nanobodies used in diagnosis will demonstrate infinite advantages. In our previous studies, Nanobodies against human procalcitonin (PCT) were successfully applied to the basis of signal amplification of CdTe@SiO2/NbII detection [21]. The PCT could respond as low as 3.4 pg/mL and showed very high sensitivity. Additionally, we have developed the human prealbumin (PA) Nanobody-based flow injection chemiluminescence immunoassay for the detection of PA with a limit of 0.01 μg/L [22].

**Scheme 1:**
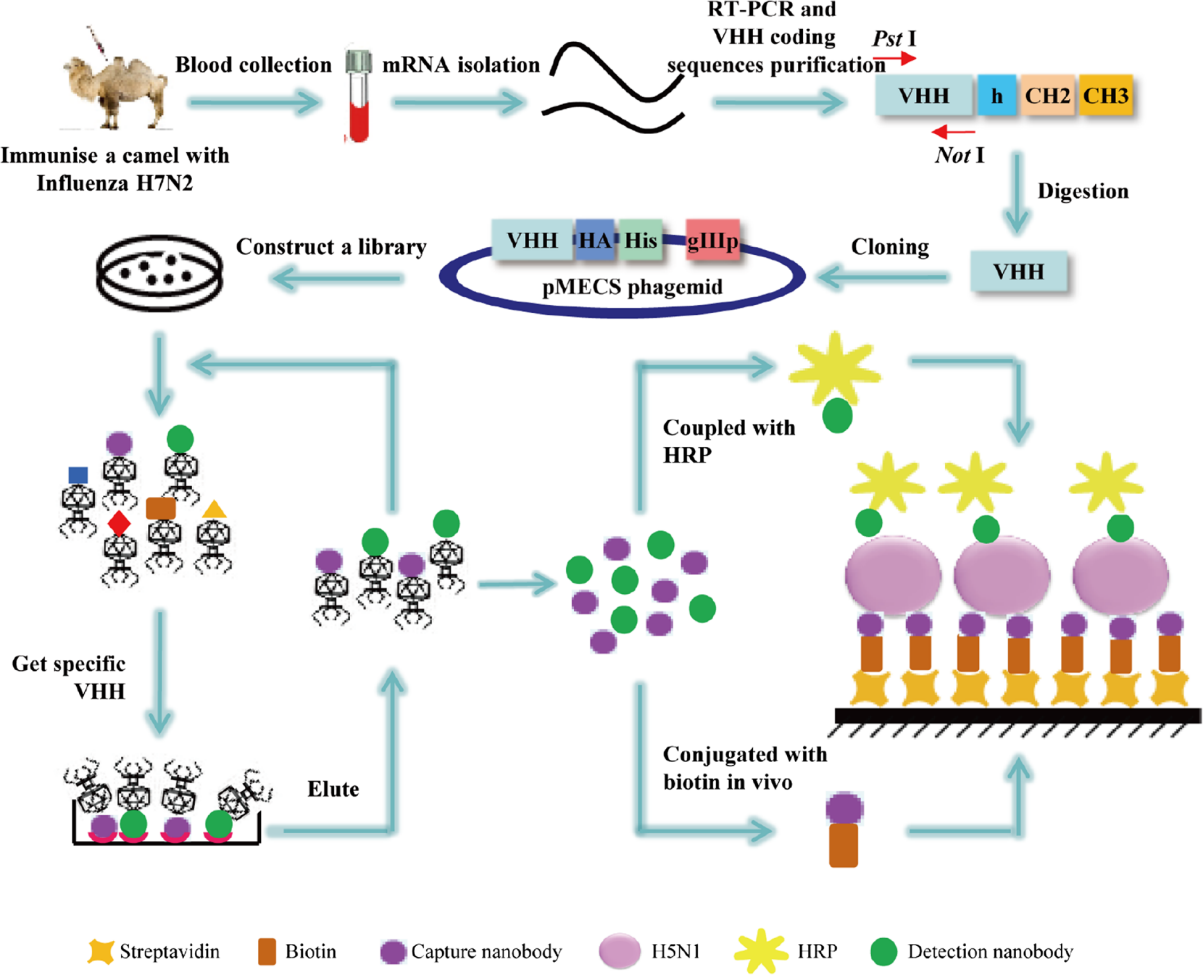
**Schematic illustration of strategies to select the H5N1-specific VHHs and to apply the detection functions.** The H5N1-specific VHH genes were selected from an immunized dromedary. Nanobodies identified different epitopes were used to couple with biotin or HRP for detecting. Biotinylated Nanobody was directionally captured by streptavidin in microtiter plate. The attachment of H5N1 virus was followed by the attachment of biotinylated Nanobody onto streptavidin. Another Nanobody conjugated with HRP was used as a detector. The measurements were taken at absorbance of 450 nm.

In this study, we have successfully constructed an immune phage display VHHs library against influenza H5N1 virus with the size of 5 × 10^8^ colonies. The VHHs recognized two different epitopes of H5N1 virus particle were used for biosensor detection system. As shown in Scheme 1, the biotinylated Nanobody was captured directionally by streptavidin coated on ELISA plates and another HRP-conjugated Nanobody was used as a detector. This streptavidin-biotin-based directional double Nanobody sandwich ELISA to detect the serum samples of H5N1 showed high sensitivity than conventional ELISA methods.

## Methods

### Reagents and materials

The influenza H5N1 virus used in this study was obtained from the Shanghai Institute of Biological Products (China). Freund’s complete adjuvant and Freund’s incomplete adjuvant were purchased from Sigma-Aldrich (USA). Fast Track 2.0 Kit was provided by Invitrogen (USA) and Oligo_dT_ primer was obtained from Thermo Scientific (USA). Restriction enzymes *Pst* I, *Not* I, *Nco* I and *BstE* II were provided from NEB (USA). The mouse anti-HA tag antibody was purchased from Covance (New Zealand). The anti-mouse IgG-alkaline phosphatase, bis phosphate and His-select column were purchased from Sigma-aldrich (USA). The D-biotin and horseradish peroxidase (HRP) were purchased from Bio Basic Inc. (China). The Streptavidin Mutein Matrix was purchased from Roche (Switzerland). The microtiter plate was purchased from Thermo Scientific NUNC (Denmark). The BeaverNano™ Streptavidin Matrix Coated 96-Well plates were provided by BeaverNano technology company (China). The VCSM13 helper phages, TG1 cells, WK6 cells, plasmid pBAD and plasmid pBirA were obtained from Prof. Serge Muyldermans’s lab (Laboratory of Cellular and Molecular Immunology, Vrije Universiteit Brussel, Belgium).

### Immunization and construction of the Nanobody library

A healthy young Bactrian camel (*Camelus bactrianus*) was immunized with inactivated influenza H5N1 virus (1 mL, 100 μg) mixed with an equal volume of Freund’s complete adjuvant for the first time, and with the same volume of Freund’s incomplete adjuvant for the next five times [23]. The camel was injected for once a week to stimulate antigen-specific B cells to express heavy chain antibodies. After the last injection, peripheral blood lymphocytes (PBLs) were extracted from 100 mL blood sample for library construction. All camel experiments were performed according to guidelines approved by the Southeast University.

For library construction, total mRNA was extracted from about 10^7^ lymphocytes and 40 μg of mRNA were used to synthesize the cDNA with the oligo_dT_ primer. To avoid contamination with the VH genes, the variable regions of the VHH were amplified by nested PCR. The first PCR was performed using the first-strand cDNA as template with the primers CALL001 and CALL002 [24]. This protocol consisted of an initial denaturation step at 94°C for 7 min, followed by 30 cycles of (94°C for 1 min, 55°C for 1 min, and 72°C for 1 min) and a final extension step at 72°C for 10 min. The PCR products consisting of ~700 bp fragments were purified by agarose gel electrophoresis and used as template for a second PCR. The primers framework-1 and framework-2 are used to amplify the Nanobodies repertoire and the final products of ~400 bp were extracted by agarose gel purification [25]. The purified second PCR product fragments were ligated into the phagemid pMECS after digestion with the restriction enzymes *Pst* I and *Not* I, and then electro-transformed into competent *E.coli* TG1 cells. The transformants were cultured on solid 2 × YT medium containing 2% glucose and 100 μg/mL ampicillin at 37°C overnight. The capacity of the constructed library was measured by the number of colonies after a gradient dilution. Then examine the insertion rate of the constructed library by PCR amplification.

### Library screening by phage display

The Nanobodies against H5N1 virus were selected by phage display. The VHHs were selected on their target coated proteins and enriched by three consecutive rounds of bio-panning, with the infection of VCSM13 helper phages. Inactivated influenza H5N1 virus (20 μg) was coated onto the microtiter plates at 4°C overnight. After blocking with 0.1% casein in phosphate-buffered saline (PBS) for 2 hours, the microtiter plates incubated with phage-displayed VHHs in PBS for 1 h at room temperature. The specific phages were eluted with 100 mM triethylamine, for 10 minutes at room temperature, and then immediately neutralized with 1.0 M Tris–HCl (pH 7.4). Then, the eluted phages were plated at various dilutions and incubated with TG1 cells for infection. The helper phagesVCSM13 were added to rescue the phages. This process represented one round of panning and the generated phage particles were used in the next round of panning. After three rounds of panning, the H5N1-specific phages were enriched gradually.

95 individual colonies were randomly selected from each round of panning and cultured in TB medium with 100 μg/mL amplicillin. The Nanobodies were expressed by induction with 1 mM Isopropyl β-D-1-Thiogalactopyranoside (IPTG). The positive clones expressing H5N1-specific Nanobodies were identified by performing a periplasmic extract ELISA (PE-ELISA). The supernatants of cells collected after an osmotic shock were transferred into the microtiter plate wells, previously coated with the H5N1 virus. Then mouse anti-HA tag antibody was added and incubated for 1 h, followed by a similar incubation with anti-mouse IgG-alkaline phosphatase for 1 h. After washing the microtiter plates with PBST (PBS with 0.05% Tween-20), the chromogenic solution containing Bis phosphate (pNPP) was added and after a few minutes the absorbance at405 nm was read. Finally, the positive clones were sequenced and classified into different families based on the diversity of the amino acids sequence in the CDR3 region (complementary determining region).

### Expression and purification

The identified VHH genes of positive clones were electro-transformed into *E.coli* WK6 cells to express Nanobodies. The cells were cultured in TB medium supplemented with 0.1% glucose, ampicillin (100 μg/mL) and 2 M MgCl_2_ at 37°C. Once the optical density reached 0.6-0.9, the cultures were induced with the addition of 1 mM IPTG and incubated at 28°C overnight. The periplasmic extract proteins were released by osmotic shock and purified by immobilized metal affinity chromatography (IMAC) using NI-NTA superflow sepharose columns with gradient concentrations of imidazole in PBS solution. The purified Nanobodies were analyzed by SDS-PAGE and dialyzed in PBS buffer.

### Specificity detection

To detect the specificity of the purified Nanobodies, several kinds of avian influenza virus were used for testing via ELISA assay. 5 μg/mL of each subtype of influenza virus were coated onto microtiter plates overnight at 4°C and blocked with 1% BSA at room temperature for 2 h. 10 μg/mL Nanobodies were added and incubated at room temperature for 1 h. The detection steps were performed by using the mouse anti-HA tag and the rabbit anti-mouse IgG-alkaline phosphatase conjugated antibodies were the same as the ones performed during the periplasmic extract ELISA described above.

### The Nanobody biotinylatedin vivo and conjugated with HRP

The genes encoding the H5N1-specific VHH were sub-cloned into the plasmid pBAD by *Nco* I and *BstE* II restriction endonucleases. The recombinant plasmids were co-transfected into WK6 cells with another plasmid, the pBirA plasmid. The VHH-BAD fusion proteins were induced with 1 mM IPTG followed by the addition of 50 μM D-biotin to the medium for 30 min. The periplasmic proteins were extracted performing the osmotic shock protocol and the biotinylated Nanobody was purified by Streptavidin Mutein Matrix and eluted with a 6 mM D-biotin solution.

To make the Nanobodies play a detecting role, horseradish peroxidase (HRP) was coupled to them. The coupling was performed by adding 0.1 M fresh NaIO_4_ into 5 mg/mL HRP for 30 min at 4°C followed by the addition of 2.5% ethylene glycol and incubation for 30 min at room temperature. Next 1 mg of H5N1-specific Nanobodies were added and incubated overnight at 4°C. After the overnight incubation sodium borohydride (5 mg/mL) was added for 3 h at 4°C, followed by dialysis into PBS. To check the efficiency of enzyme labeled Nanobodies, 5 μg/mL of H5N1 antigen were coated and 10 μg/mL of Nanobodies coupled with HRP were used as detectors to perform the ELISA experiment. Chromogenic reactions were proceeded with 3, 3′, 5, 5′-Tetramethylbenzidine for 10 minutes and stopped by addition of 2 M H_2_SO_4_, the absorbance was measured at 450 nm.

### Double Nanobodies sandwich ELISA

Directional double Nanobodies sandwich ELISA was performed based on streptavidin-biotin system. 1 μg/mL of biotinylated Nanobodies were added into streptavidin matrix coated plate and incubated at room temperature for 1 h. Several concentrations of H5N1 virus (0, 10, 50, 100, 500, 1000, 5000 ng/mL) were added for 2 h after blocked with 5% BSA for 1 h. The Nanobodies conjugated with HRP were diluted with 5% BSA to 1 μg/mL and incubated with captured H5N1 virus for 1 h. Finally, the measurements of chromogenic reaction were read at 450 nm.

For the undirectional double sandwich ELISA, unbiotinylated Nanobody was used as a capturing Nanobody and coated onto a conventional microtiter plate. Seven concentrations of H5N1 virus (0, 10, 50, 100, 500, 1000 ng/mL) were used to test this system. After blocking, antigen reaction and Nanobody-HRP reaction, the chromogenic reaction was measured at 450 nm.

### Recovery test

The directional sandwich ELISA was used for H5N1 virus detection in human serum samples. 5 concentrations of H5N1 virus in linear range (50, 100, 250, 500, 1000 ng/mL) were added into human serum sample to test the directional system. Each concentration was replicated three times.

## Results

### Immunized VHH library construction

The VHH library was constructed after immunization of a healthy camel with the H5N1 virus for 7 weeks. These H5N1 viruses were isolated from eggs and inactivated by gamma radiation. The total IgG titer of serum reached to 1:1000 after the last immunization and it was considered that it had raised good immunogenic response. The nested PCR assured the accuracy and feasibility of the whole reaction. The first PCR products contained the 700 bp fragments of the VH-CH2 exons, and were the templates for the second PCR that generated products of 400 bp fragments for the VHH exons.

**Figure 1:**
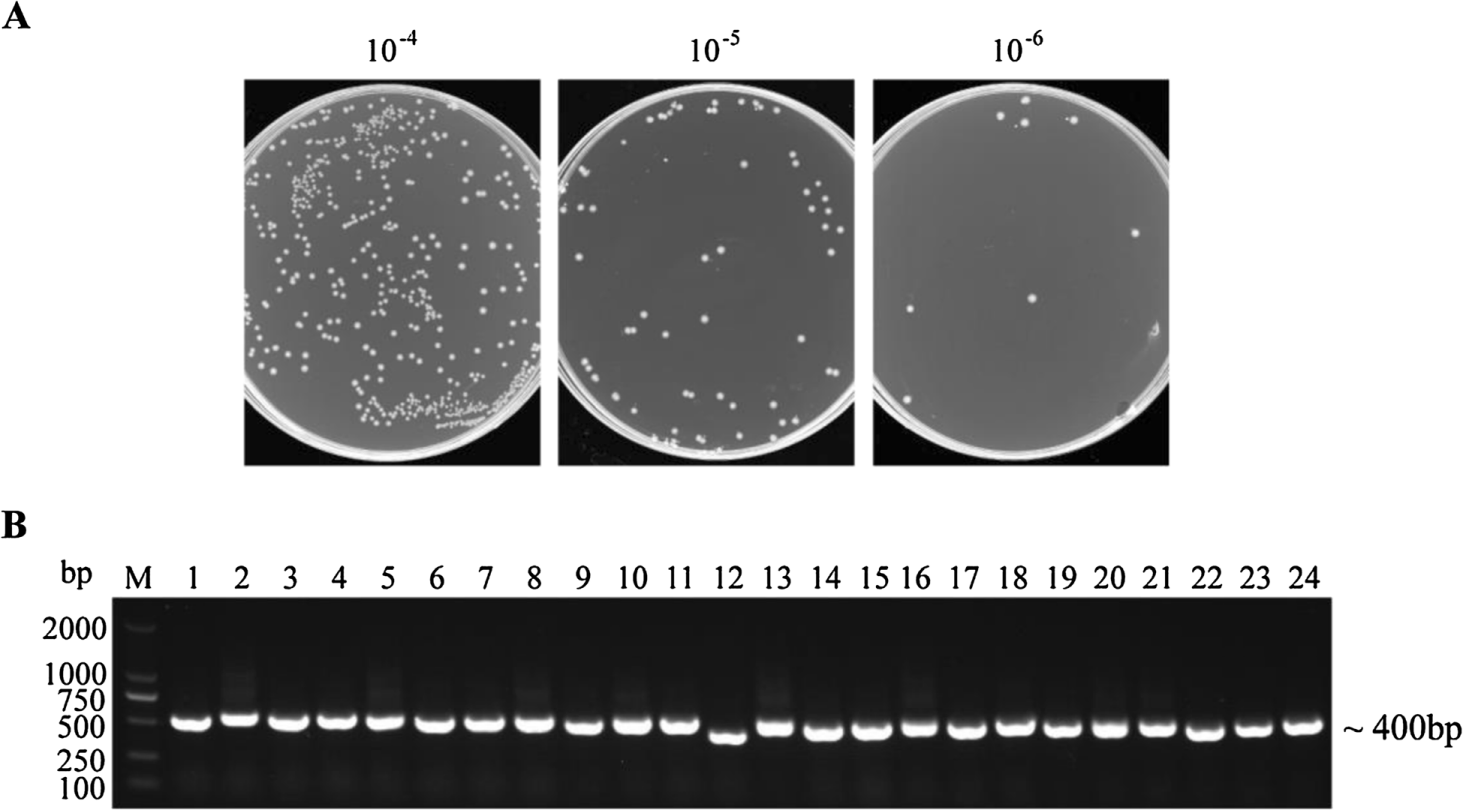
**Library construction. (A)** The size of the library was determined by counting the number of clones after gradient dilution and the library size was 5 x 10^8^ cfu/mL. **(B)** 24 colonies were randomly selected to estimate the correct insertion rate by performing PCR. The size of products was approximately 400 bp.

To construct the library, *Pst* I and *Not* I sites were introduced in the 5′ and 3′ ends of the VHH fragments, respectively. 4.8 μg of digested VHH fragments and 16 μg of linearized pMECS vector were used for ligation. The connection products were transformed into TG1cells by 30 electroporation transformations. The titer of this Nanobody library against H5N1 was calculated by counting the number of colonies in gradient dilution plates (Figure 1A). The titer reached to 5 × 10^8^ cfu/mL meaning that it was highly possible to obtain Nanobodies with high specificity and sequence diversity. Another parameter of quality control from the library is to check the insertion rate. 24 colonies were randomly chosen for PCR analysis and the percentage of library insertion rate was 100% as shown in Figure 1B. These results indicated that we have successfully constructed an immune phage display library with high quality for the following H5N1-specific Nanobodies selection.

### Library screening and selection of H5N1-specific Nanobodies

**Figure 2:**
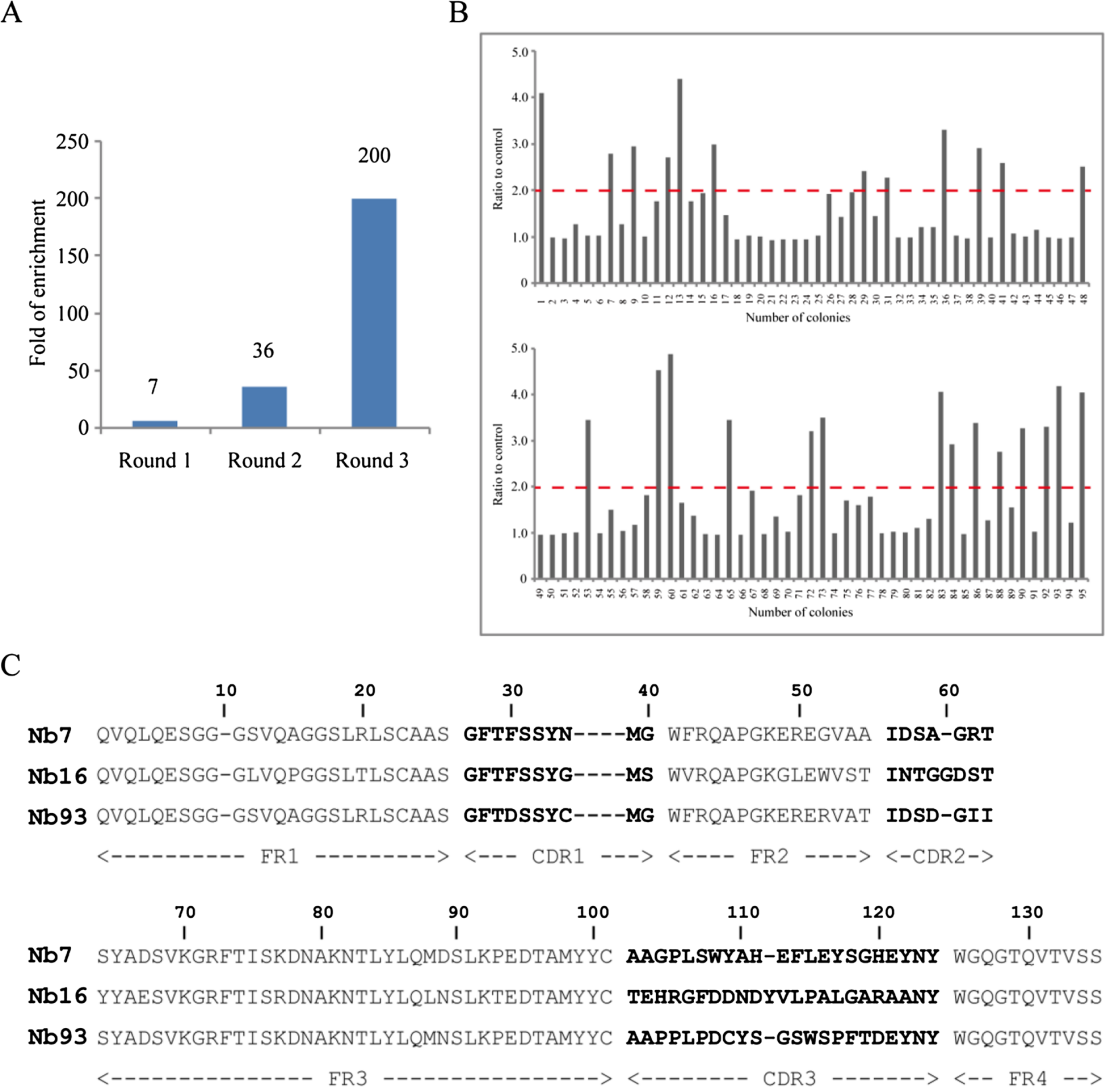
**Nanobodies against H5N1 were selected by phage display library. (A)** The enrichment was detected and H5N1-specific VHHs were enriched about 200-fold after three rounds of panning. **(B)** Periplasmic extract ELISA for 95 clones for detecting and 26 clones were identified as the positive clones. The ratio higher than 2 was considered as positive. **(C)** Three kinds of different amino acid sequences of anti-H5N1 VHHs were identified.

Bio-panning was performed to isolate the H5N1-specific Nanobodies after a high quality phage display library was constructed. 2 × 10^11^ phages were collected from the library and used for incubation with H5N1 virus particles. The phages expressing H5N1-specific VHHs were enriched by consecutive rounds of bio-panning on H5N1. Compared with the negative control, the specific VHHs enriched 200-fold after three rounds of panning (Figure 2A).

Then, 95 colonies randomly picked up from each round were screened for influenza H5N1 virus recognition by periplasmic extraction followed by ELISA. The cultured cells were disrupted by osmosis in our protocol, allowing the Nanobodies to be collected in the supernatant and followed by incubation with antigen. Thus, we called this assay a periplasmic extraction ELISA (PE-ELISA). As shown in Figure 2B, 26 colonies were found to specifically bind H5N1 virus but not the negative blank control. Here we often identified the positive clones whose binding ratios more than 2. According to our previous studies [21,22,26], high enrichment within the 2–4 rounds of panning for common antigen will benefit and the majority of specific VHHs could be selected from the second and third rounds [27].

After identified positive clones by PE-ELISA, the sequence of 26 clones was analyzed and divided into three families. Their paratope (CDR3 region) amino acid sequences differed by some amino acids as shown in Figure 2C.

### Expression of the Nanobodies and specificity detection

**Figure 3:**
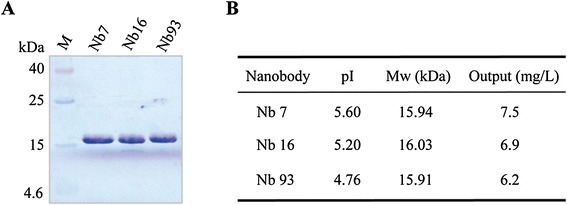
**Purification of H5N1-specific Nanobodies. (A)**Three Nanobodies encoded by different sequence were purified by immobilized metal affinity chromatography (IMAC) using a His-Select matrix. These Nanobodies were detected by coomassie brilliant blue stained SDS-PAGE. **(B)** Isoelectric point, molecular weight and output of three Nanobodies.

The Nanobodies expressed in WK6 *E.coli* cells were fused with *pelB* leader signal sequence and the soluble Nanobodies were further purified by Ni-NTA Superflow Sepharose columns. Sodium dodecyl sulfate polyacrylamide gel electrophoresis (SDS-PAGE) analysis demonstrated the high quality of the Nanobodies, with more than 90% purity obtained (Figure 3A). Moreover, milligram quantities of production were yielded (Figure 3B).

**Figure 4:**
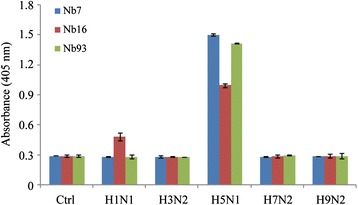
**Specificity detection of three Nanobodies against H5N1 by ELISA.** Different subtypes of influenza viruses were coated onto microtiter plates and the Nanobodies were added to incubate with them. After the reaction with the mouse anti-HA tag antibody and then with the rabbit anti-mouse IgG-alkaline phosphatase, the chromogenic solution containing bis phosphate were added and the absorbance was measured by an ELISA reader at 405 nm. The values were the means of three replicates. Nb7 and Nb93 only recognized H5N1 virus but not other subtypes.

The specificity of these Nanobodies was demonstrated by ELISA. The results in Figure 4 showed that Nanobody NO.16 (Nb16) not only specifically binds to H5N1 but also to H1N1 viruses. Nanobody NO.7 (Nb7) and NO.93 (Nb93) were shown to bind specifically to the H5N1 virus but not to the other subtypes of influenza A viruses. Based on these results, Nb7 and Nb93 were used for the Nanobody-pairing assay. The Nanobody-pairing assay was performed from these three H5N1-specific Nanobodies. It turned out that Nb7 and Nb93 could greatly combine with H5N1 virus particles for the further diagnostic application based on sandwich ELISA.

### Nanobody biotinylation based on in vivo assay

In order to execute Nanobody biotinylation, VHH genes were sub-cloned into pBAD17 vector containing a Biotin Acceptor Domain (BAD), at the downstream of the VHH sequence. Then, the recombinant plasmids were co-transformed into WK6 cells with another plasmid pBirA (encoding biotin protein ligase) to express biotinylated Nanobodies. This method labeled Nanobody with biotin in vivo will not disrupt the combination between Nanobodies and antigen. Nb7 was chosen for conjugation with biotin and another one, Nb93, was used as a detector to couple with HRP. The biotinylated Nanobodies were extracted from the periplasm of cells by osmotic shock as described previously and further purified with only 200 μL Streptavidin-Mutein Matrix repeated seven times. It was produced at a final yield of 2.1 mg/L culture.

### Streptavidin-biotin-based directional double Nanobodies sandwich ELISA

**Figure 5:**
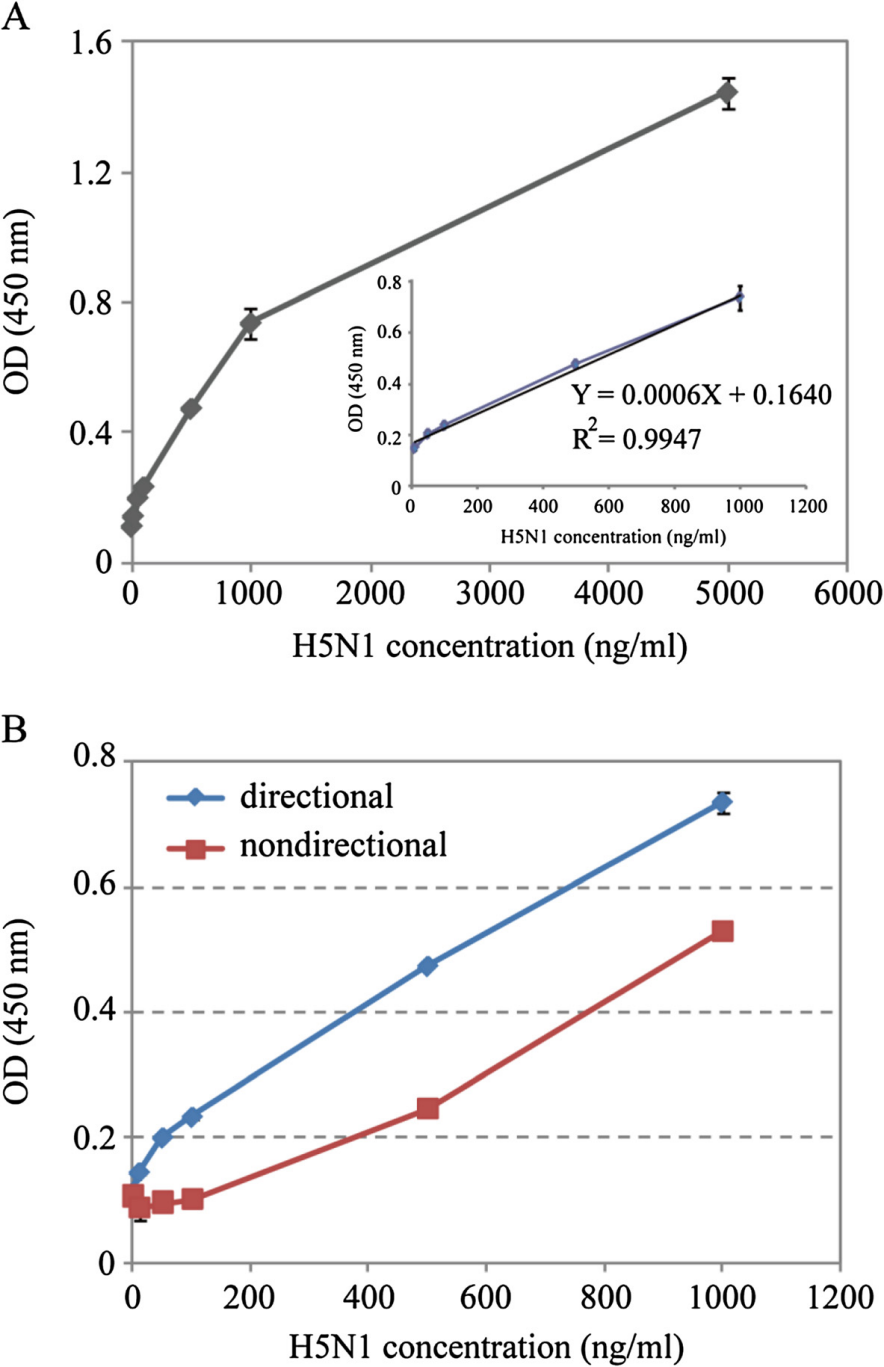
**Detection of H5N1 virus by directional sandwich ELISA and comparison with the conventionally undirectional ELISA. (A)** Serial concentrations (0, 10, 50, 100, 500, 1000, 5000 ng/mL) of H5N1 antigen were used for detection. The linear relationship in the range from 50 to 1000 ng/mL. The linear equation was calculated as Y = 0.0006 X + 0.1640 with an acceptable correlation coefficient of 0.9947 (R^2^). The data represent mean ± standard deviation from triplicate measurements. **(B)** The concentration of H5N1 from 0 to 1000 ng/mL to test directional and undirectional sandwich ELISA system. Error bars are standard deviations of three measurements.

The result of this streptavidin-biotin-based directional double Nanobodies sandwich ELISA was shown as Figure 5A. Under the optimal conditions, seven gradient concentrations (from 0 to 5000 ng/mL) were performed in our detection. The absorbance at 450 nm displayed a good linearity with the concentrations of H5N1 virus in the range from 50 to 1000 ng/mL. The linear equation was calculated as Y = 0.0006X + 0.1640 with an acceptable correlation coefficient of 0.9947 (R^2^). The detection limit of this method for H5N1 virus was 14.1 ng/mL. However, as shown in Figure 5B, the unbiotinylated Nanobodies directly coated in microtiter plate to capture H5N1 virus and detected by Nb-HRP did not show a good linearship from 50 to 1000 ng/mL. Only the concentration of H5N1 virus with 500 ng/mL can be detected. Overall, the directional system showed an excellent sensitivity than the undirectional ELISA.

Finally, sample matrix effects were examined by sample dilution with buffer. Serum samples were added with H5N1 virus at different concentrations (50–1000 ng/mL) and assayed by directional ELISA. The recoveries of H5N1 virus from human serum samples are shown in Table 1. The average recoveries from triplicate measurements ranged from 94.58% to 114.51% with a relative standard deviation (RSD) less than 6.5%. This result indicated that directional double Nanobodies sandwich ELISA based on streptavidin-biotin system could effectively detect influenza H5N1 virus in real samples. It may provide promising diagnostic materials for clinical application.

**Table 1:** **Streptavidin-biotin-based directional ELISA for H5N1 detection in serum samples**

**Sample**	**H5N1 concentation (ng/ml)**		**Recovery**	**RSD**
	**Added**	**Found**	**(%)**	**(%)**
1	50	47.29	94.58	4.24
2	100	114.51	114.51	4.13
3	250	253.18	101.27	6.23
4	500	488.65	97.73	2.72
5	1000	952.80	95.28	2.14

## Discussion

In our study, the VHH fragments encoding three kinds of H5N1-specific Nanobodies in phage plasmid pMECS were directly transformed into WK6 *E.coli* cells. Unique properties made these cells cannot suppress the amber stop codon between gene III and VHH from pMECS [28]. Moreover, the Nanobodies expressed in WK6 *E.coli* cells were fused with *pel*B leader signal sequence [28]. These Nanobodies with *pel*B leader signal peptide will be only expressed in the periplasmic space. Thus, all the Nanobodies were present in the supernatant when the cells were disrupted by osmostic shock. However, the conventional method is to sub-clone the VHH fragment from the phagemid into an expression plasmid [27]. The present method is more convenient that we do not necessary to do sub-cloning step.

It is well known that the binding of biotin to streptavidin is one of the strongest non-covalent interactions known in nature [29]. The complex of biotin-streptavidin can resist some severe environments such as organic solvents, proteolytic enzymes and extremes of temperature and pH [30]. Therefore, this system can be used extensively in molecular biology and bionanotechnology. In immune detection systems, streptavidin is usually directly immobilized on the sensor chip, magnetic bead, electrode and so on. However, these methods usually need high quality of both materials and instruments. In order to make a rapid, inexpensive and accurate detection of H5N1, we choose the microtiter plate to perform this assay. As shown in Scheme 1, biotinylated Nanobodies were directionally captured by streptavidin which was previously coated on a microtiter plate. High density of streptavidin can bind more biotinylated Nanobodies. These Nanobodies evenly aligned and emerged antigen binding site. When H5N1 virus was recognized and captured by biotinylated Nanobody, another Nanobody coupled with HRP was used for detection.

Avian influenza H5N1 is a potential highly pathogenic virus threatening to human health. However, current clinical biochemical diagnosis for influenza virus are still flawed, and the diagnostic kits of H5N1 commercially available are mainly based on the traditional monoclonal antibody to hardly meet requirements of the clinical application. The limitation of detection using this method is lower than the reported research using traditional monoclonal antibody [16], Moreover, current commercial diagnostic kits for influenza viruses from Binax, Inc. showed the LOD were approximately 100 ng/mL (http://www.biomedix.com.br/bulas/binax). However, the novelty directional ELISA based on biotinylated Nanobody showed the superiority than conventional undirectional ELISA. Moreover, Nanobodies used in this study are highly specific, high-yield and stable. These advantages will be made a lower cost and highly sensitivity for the promising diagnostic kits.

## Conclusions

In this study, we successfully selected Nanobodies against H5N1 virus from a high quality library and developed a directional, rapid and sensitive immunoassay for H5N1 virus detection based on Nanobodies. Directional double Nanobody sandwich ELISA based on streptavidin-biotin system showed higher sensitivity than conventionally undirectional sandwich ELISA. It demonstrated that the proposed detection system is an alternative way to enable rapid, low-cost and highly specific detection of H5N1 virus and may be easily applied for the detection of other subtypes of influenza virus or any other proteins.
